# Investigation of the adaptation of *Lactococcus lactis* to isoleucine starvation integrating dynamic transcriptome and proteome information

**DOI:** 10.1186/1475-2859-10-S1-S18

**Published:** 2011-08-30

**Authors:** Clémentine Dressaire, Emma Redon, Christophe Gitton, Pascal Loubière, Véronique Monnet, Muriel Cocaign-Bousquet

**Affiliations:** 1 Université de Toulouse; INSA, UPS, INP; LISBP, 135 Avenue de Rangueil, F-31077 Toulouse, France; 2 INRA, UMR792 Ingénierie des Systèmes Biologiques et des Procédés, F-31400 Toulouse, France; 3 CNRS, UMR5504, F-31400 Toulouse, France; 4 INRA, Unité de Biochimie Bactérienne, UR477, F-78350 Jouy en Josas, France

## Abstract

**Background:**

Amino acid assimilation is crucial for bacteria and this is particularly true for Lactic Acid Bacteria (LAB) that are generally auxotroph for amino acids. The global response of the LAB model *Lactococcus lactis* ssp. *lactis* was characterized during progressive isoleucine starvation in batch culture using a chemically defined medium in which isoleucine concentration was fixed so as to become the sole limiting nutriment. Dynamic analyses were performed using transcriptomic and proteomic approaches and the results were analysed conjointly with fermentation kinetic data.

**Results:**

The response was first deduced from transcriptomic analysis and corroborated by proteomic results. It occurred progressively and could be divided into three major mechanisms: (i) a global down-regulation of processes linked to bacterial growth and catabolism (transcription, translation, carbon metabolism and transport, pyrimidine and fatty acid metabolism), (ii) a specific positive response related to the limiting nutrient (activation of pathways of carbon or nitrogen metabolism and leading to isoleucine supply) and (iii) an unexpected oxidative stress response (positive regulation of aerobic metabolism, electron transport, thioredoxin metabolism and pyruvate dehydrogenase). The involvement of various regulatory mechanisms during this adaptation was analysed on the basis of transcriptomic data comparisons. The global regulator CodY seemed specifically dedicated to the regulation of isoleucine supply. Other regulations were massively related to growth rate and stringent response.

**Conclusion:**

This integrative biology approach provided an overview of the metabolic pathways involved during isoleucine starvation and their regulations. It has extended significantly the physiological understanding of the metabolism of *L. lactis* ssp. *lactis*. The approach can be generalised to other conditions and will contribute significantly to the identification of the biological processes involved in complex regulatory networks of micro-organisms.

## Background

Free living bacteria have to adapt to various adverse environmental conditions such as nutrient deprivation. To survive and develop in such challenging environments, they have evolved a wide set of adaptation responses involving complex regulatory networks that are not yet completely understood. The adaptation of Lactic Acid Bacteria (LAB) to amino acid starvation is particularly interesting. The LAB model *Lactococcus lactis* ssp. *lactis* is characterized by numerous amino acid auxotrophies. The strain IL1403 [1], used in this study, displays seven such amino acid auxotrophies [2]. It is indeed known to have lost its ability to synthesize some amino acids such as histidine [3] or branched chain amino acids (BCAA) [4] due to a combination of both punctual mutations and gene deletions. In LAB, amino acids are required for protein synthesis and more largely for cell physiology [5-7]. Extensive attention has been paid to amino acid transport and assimilation [8,9] as well as bacteria ability to produce flavor compounds through their degradation [5,6]. However, to date, no global study of how amino acid starvation provokes modifications to cell metabolism is available.

Genome wide analyses of amino acid starvation have been reported for *Bacillus subtilis *[10], *Bordetella pertusis *[11] and *Escherichia coli *[12] revealing an unexpectedly complex regulation pattern. The general mechanism of the stringent response, which is mediated by the RelA protein and (p)ppGpp alarmone [13-16], was seen to be involved. Other regulators such as stress-related alternative sigma factor(s), the carbon catabolite repressor or even other unidentified regulators were also assumed to interfere. Regulations during amino acid starvation are not well understood in *L. lactis* ssp. *lactis*. This bacterium does not have any stress-related alternative sigma factors [1]. The stringent response mechanism, investigated in *L. lactis* ssp. *lactis* by transcriptomic approach after norvaline induction [17], is potentially involved. The well known nitrogen metabolism regulator CodY [18], whose activity was shown to be mediated by the branched chain amino acids (BCAA) pool [18,19], is also expected to be involved. Since such starvation is always accompanied by modified growth rates, it might be assumed that other general mechanisms associated to growth rate regulation [17,20], would be involved.

The aim of this paper is both to provide a complete overview of the adaptative response of *L. lactis* ssp. *lactis* to isoleucine starvation and to decipher its regulatory network. Isoleucine is the second most consumed amino acid during growth of *L. lactis* ssp. *lactis *[1] due to its high abundance in proteins and might therefore be expected to play a central role in nitrogen metabolism. Here, isoleucine starvation was gradually imposed through the natural consumption of isoleucine present in the medium. The dynamic adaptation was analyzed through transcriptome and proteome measurements. This integrative approach revealed a complex regulatory network interrelating carbon and nitrogen metabolism.

## Methods

### Organism, growth conditions and fermentation analytical methods

*Lactococcus lactis* ssp. *lactis* IL1403, whose genome has been entirely sequenced [1], was used throughout this study. Bacteria were grown in modified chemically defined medium [21,22] with ten-fold reduced BCAA concentrations ([isoleucine] = 150 µM, [leucine] = 360 µM, [valine] = 280 µM). At least four independent repetitions of the culture were analyzed. Cultures were performed at 30 °C under anaerobic conditions in a 2 L fermentor (Setric Génie Industriel, Toulouse, France) with an agitation speed of 250 rpm. The pH was maintained at 6.6 by automatic addition of KOH 10 N. Bacterial growth was estimated by spectrophotometric measurement at 580 nm (1 OD unit is equivalent to 0.3 g.L^-1^). Inoculation was realized with exponential phase cells from pre-cultures grown in the same medium so as to obtain an initial OD_580_ of 0.08.

Glucose and fermentation products (lactate, formate, acetate and ethanol) concentrations were measured every 20 minutes by high-performance liquid chromatography as previously described [23]. Amino acids concentrations were determined with an AminoQuant 1090 high-performance liquid chromatography (Hewlett Packard, San Fernando, CA). After precipitation of proteins at 4 °C by addition of four volumes of methanol, samples were chemically modified (derivatization in presence of 3-mercaptopropionic acid by ortho-phtalaldehyde and 9-fluorenylmethyl chloroformate for primary and secondary amino acids respectively), separated with a C18 column and detected by spectrophotometry at 338 and 262 nm.

Instantaneous specific rates for biomass, glucose and lactate were calculated at each time of the fermentation by the time derivate of each concentration profile divided by the corresponding biomass concentration.

### Transcriptomic analyses

**Figure 1:**
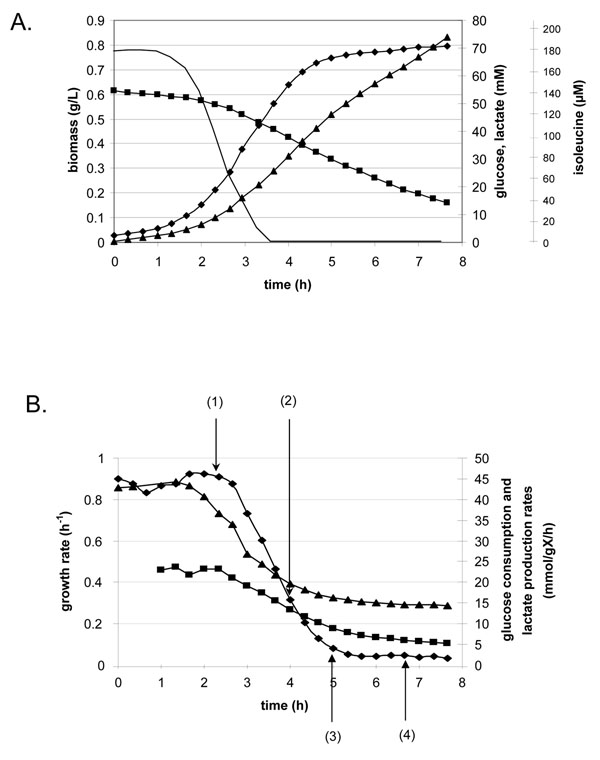
**Kinetic profile of *L. lactis* IL1403 during isoleucine starvation (average of at least four independent repetitions)** A. Concentrations of biomass (♦), glucose (■), lactate (▲) and isoleucine (black line). B. Specific rates of growth (♦), glucose consumption (■) and lactate production (▲). The numbered arrows indicate the sampling for both proteome and transcriptome analysis. (1): exponential phase corresponding to the reference; (2): 20 minutes of starvation; (3): 1.34 h of starvation; (4): 3 h of starvation.

Membrane spotting and analytical support were provided by the Biochips Platform (Genopole Toulouse, France). Cell samples were harvested from culture in exponential growth phase and after 20 min, 1.34 h and 3 h of amino acid starvation, corresponding respectively to 2.7 h, 4 h, 5 h and 6.66 h of culture (see Figure 1B). Cell sampling and lysis and total RNA extraction were performed as previously described [24]. Briefly, a volume of culture corresponding to 6 mg (dry weight) of cells were frozen immediately in liquid nitrogen, RNA was extracted with glass beads at 4 °C, quantified at 260 nm and RNA quality was controlled both on electrophoresis agarose gel in denaturing conditions and on Agilent Bioanalyzer^®^. Gene expression was measured by using nylon arrays displaying 1948 ORFs of *L. lactis* IL1403 out of the 2310 ORFs identified in the genome [1]. The PCR set specific for *L. lactis* IL1403 was provided by Eurogentec. A constant amount of 10 µg of total RNA was retro-transcribed. Synthesis of radio-labeled cDNA, nylon array hybridization and washings were performed as previously described [24]. Membranes were exposed to a phosphoimager screen for three days and scanned with a phosphofluoroimager (Storm 860, Molecular Dynamics, Maryland, USA). Hybridization signals were quantified, assigned to gene names, and statistically treated with the Bioplot software (developed by S. Sokol in the Biochips Platform, Toulouse, see http://biopuce.insa-toulouse.fr). For each condition, three repetitions were performed with independent cultures, extractions, labelling and hybridizations. After background removal, raw data, available online in Gene Expression Omnibus (GEO, http://www.ncbi.nlm.nih.gov/geo/) database (series accession number: GSE12962, the already available platform GSE4872 also corresponding to isoleucine starvation was not used because biological replicates were not independent), were normalized by the mean intensity of the corresponding membrane. Expression ratios were calculated after 20 min, 1.34 h and 3 h of isoleucine starvation using the exponential phase as a reference. The statistical significance of expression ratios were evaluated using False Discovery Rate (FDR) calculations. Genes with an expression ratio associated to a FDR lower than 10 % were considered differentially expressed. Each of these genes displayed individual Student p-value inferior to 0.05. In order to determine expression changes at the level of the functional (sub)categories (global tendencies), over- or under-expressed gene enrichments in the different groups were calculated with the Wilcoxon test as previously described [17]. A Wilcoxon p-value lower than 0.05 was considered to be significant.

### Quantitative RT-PCR

In order to confirm transcriptomic results RT-PCR measurements were performed in exponential phase and after 1.34 h of isoleucine starvation. Nine genes were chosen so as to cover a wide range of functional categories and also a large range of transcriptomic changes. *adhE*, *glpF1* and *ptnAB* are involved in carbon metabolism, *busAB*, *ilvD* and *optC* in nitrogen metabolism, *rplM* in translation, *rpo* in replication and *clpE* in adaptation mechanisms. Half of these genes were over- (*busAB*, *clpE*, *glpF1*, *ilvD*, *optC*) and under-expressed (*adhE*, *ptnAB*, *rplM*, *rpoB*). Ten µg of RNA samples extracted from cells were retro-transcribed, RNase H-treated and purified as previously described [24] except that unlabelled dCTP and only random primers were used. Primer pairs were designed with Beacon Designer 3.0 software (Premier Biosoft, Palo Alto, CA, USA) to fulfill the following criteria: length of 22 ± 2 bp, Tm of 60 ± 1 °C, GC content superior to 50 % and amplicon lengths ranging from 75 to 150 bp (Table 1). Primer specificity was controlled with Blast analyses. Real-time PCR was carried out on a Biorad-MyIQ with the IQ^TM^ SYBR^®^ supermix in 96-well plates. Five µL of diluted cDNA were added to 20 µL of PCR mixture (12.5 µL of IQ^TM^ SYBR^®^ supermix, 2 µL of each primer at 5 µM and 3.5 µL of RNase-free water). Amplification program consisted in a denaturation at 95 °C for 3 min followed by 40 cycles at 95 °C for 15 s and 60 °C for 45 s. Fluorescence was measured during each annealing step and melting curves were performed from 60 to 95 °C (0.05 °C.s^-1^) to validate the specificity of PCR reaction. In each run, three dilutions of cDNA were analyzed to determine the PCR efficiency and negative controls were included. Three independent measurements were performed for each gene and culture condition. The threshold cycle values (C_T_) were determined with a baseline set manually at 135 relative fluorescence units (baseline above background and corresponding to PCR efficiencies ranging from 90 to 110 %). Results were analyzed using the comparative critical threshold method (ΔΔC_T_) in which the amount of target RNA is adjusted to an internal reference [25]. *ldh* gene, encoding lactate dehydrogenase, did not show significant expression variation in these experiments and was used as an internal reference to normalize the results. Expression ratios were expressed as 2^ΔΔ^*^C_T_^*.

**Table 1:** Primer sequences used for qRT-PCR.

Target gene	Forward primer	Reverse primer	Amplicon length (bp)
*adhE*	CTGACCCAACTTTGAGCGAAGC	AGAGCAGAACCACCACCAAGAC	94
*busAB*	TTCCAGCGGTTGCATTCTTTGG	TTCTGACTGTTGGTGGGAGTGC	84
*clpE*	GCAGCAAGCACTCAAACTCCAC	ACGACGAGCTGACTCTGTGATG	132
*glpF1*	AATGGGGTTGTCGCAGCAAATG	AGAAGCCAACTACCCAGACAGC	118
*ilvD*	CAACACAACCAGCGACTCAAGC	ATGCTGACGATTCCGACCTGAG	88
*ldh*	ATGGTGTCGCTGTAGCTCTTG	GCAGTCGCTTACGCCATATTG	110
*optC*	TTTGCATGGACCGGGATGGC	TGAGGCCAAGACGAAGTCACG	78
*ptnAB*	CTGCTGTTCAAGGTGCGATTCC	ACGATGATGCGGTTTGCTCTTG	145
*rplM*	TGGTACGTCGTCGATGCAACTG	TTTTCCACGAAGTACGCTTGCG	75
*rpoB*	TCCTGATGCAGAATGCCGTGTC	TCAGCAAGACCAAGCCAGTAGC	75

### Proteomic analyses

For each condition, three repetitions were performed with independent cultures, extractions and electrophoresis. The various step of the proteomic analysis (2-Dimensional gel electrophoresis and MS protein identification) have been previously described [20]. Raw spot volumes were normalized by the mean intensity of the corresponding gel. Only the spots containing a single protein were considered. When a protein was present in several spots of a same gel, the protein abundance corresponded to the sum of the intensity of the different spots. Proteins identified both on 4.5-5.5 and 5-6 pH ranges were considered and analyzed independently as two unique entities. The statistical significance of expression ratios was evaluated using Student test. Proteins with an abundance ratio associated to a Student p-value lower than 0.05 were considered differentially abundant. The mean FDR of all the selected proteins was lower than 20 %.

## Results and discussion

### Isoleucine starvation and kinetic adaptation of *L. lactis* ssp. *lactis*

The dynamic adaptation of *L. lactis* IL1403 to amino acid starvation was studied in batch culture. This stress was imposed progressively by the natural consumption of isoleucine during cell growth. Biomass, glucose, end products and amino acid concentrations were determined experimentally (see Methods) over the 8 h duration of the fermentation (Figure 1A). Isoleucine was exhausted at 3.66 h of culture, when glucose and biomass concentrations were respectively 41 mM and 0.56 g.L^-1^. Glutamate was lacking from the culture medium but was produced after isoleucine exhaustion; its concentration reached approximately 100 µM at the end of the culture. Proline was produced from the beginning of the fermentation while tryptophan consumption was not detected under our conditions (data not shown). All other amino acids were consumed throughout the fermentation but were still present at the end of the culture with concentrations ranging from 70 to 2500 µM (data not shown). Those concentrations were estimated to be high enough to sustain growth without isoleucine for several hours.

At the beginning of the culture, growth of *L. lactis* IL1403 was exponential with a maximum rate of 0.88 h^-1^, characteristic of a non-limited growth state on such defined media (Figure 1B). Between 3 and 5 h, as isoleucine became limiting, growth progressively decelerated. After this deceleration phase and under conditions of isoleucine depletion, a minimal growth rate was maintained at a constant but extremely low average value of 0.05 h^-1^ corresponding to a generation time of 13.9 h. This slow growth was maintained when the cells were transferred into fresh CDM medium lacking isoleucine (two successive transfers corresponding to at least 10 generations). Although *L. lactis* IL1403 was previously reported as an isoleucine auxotroph [2,4], it was shown in this study that this strain could sustain a constant growth in the absence of isoleucine, despite at an extremely low rate. Godon *et al.*[4] considered *L. lactis* IL1403 as auxotroph for an amino acid when no colony was formed after 24 h incubation on agar plate lacking this specific amino acid. They have nevertheless observed small colony formation after 3 days without isoleucine, thus agreeing with our finding that *L. lactis* IL1403 is not completely an isoleucine auxotrophic strain. Glucose consumption (q_S_) and lactate production (ν_L_) rates were high and constant during the exponential phase (22 ± 3 mmol.g^-1^.h^-1^ and 42 ± 2 mmol.g^-1^.h^-1^ respectively). Significantly lower values (7 ± 1 mmol.g^-1^.h^-1^ and 15 ± 1 mmol.g^-1^.h^-1^ respectively) were obtained in the slow growth phase (Figure 1B). In both phases, lactate production accounted for almost 90 % of the glucose consumption, indicating a constant homolactic metabolism.

### Overview of transcriptomic and proteomic responses

Both transcriptome and proteome analyses were performed during exponential phase (2.7 h of culture) and after 20 min, 1.34 h and 3 h of isoleucine starvation (respectively 4 h, 5 h and 6.66 h of culture; see Figure 1B). Three biologically independent repetitions of transcriptomic and proteomic analyses were performed for statistical treatment. Raw transcriptomic data are available online in the GEO database (series accession number: GSE12962). The list of all identified proteins with their mass spectrometry characteristics and average protein amounts with their standard deviations are provided in Additional files 1 and 2. For technical reasons, transcriptome analysis has delivered much more information than proteome (detection of 1948 genes compared to 341 proteins), as is often the case in the well documented literature [26-28]. All the transcripts and proteins considered as differentially abundant during growth kinetics, using the statistical criteria described in materials and methods are listed in Additional file 3.

**Figure 2:**
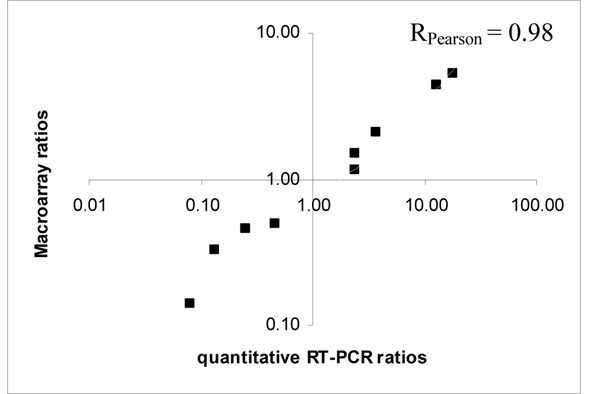
**Macroarray and qRT-PCR comparison in *L. lactis* IL1403.** Log-log scatter plot of expression ratios between exponential phase and 1.34 h of isoleucine starvation obtained by macroarray (*y*-axis) and qRT-PCR (*x*-axis) analyses for 9 genes (*adhE*, *busAB*, *clpE*, *glpF1*, *ilvD*, *optC*, *ptnAB*, *rplM* and *rpoB*).

The response of *L. lactis* IL1403 to isoleucine starvation is progressive. When compared to the exponential phase, 309, 420 and 587 genes were differentially expressed after 20 min, 1.34 h and 3 h of isoleucine starvation respectively. The general reliability of the macroarray data was confirmed experimentally by the strong correlation with qRT-PCR results (Figure 2; Pearson coefficient of 0.98, p-value = 10^-6^). Similarly, the levels of 30, 38 and 41 proteins were significantly modified while starvation occurred. Clearly, the longer the time after isoleucine starvation, the more genes and proteins were involved in the response. This shows that the dynamic approach is useful to provide the most complete description of the response.

In order to analyze transcriptomic results, global expression tendency for each functional category was estimated using the Wilcoxon test as previously described [17]. Over- or under-expressed (sub) categories are listed in Additional file 3 (Wilcoxon p-value < 0.05). For proteomic results, these enrichment calculations were not possible due to the small size of the dataset. However, individual protein changes generally confirmed transcriptomic analysis tendencies (Additional file 3).

### Three major mechanisms are involved in isoleucine starvation adaptation

The transcriptomic and proteomic responses of *L. lactis* IL1403 to isoleucine starvation (Additional file 3) can be summarized as three major mechanisms.

#### i) Negative control of major physiological activities

A general negative response was observed on the expression of genes and proteins involved in major physiological processes. The functional category "translation" was mostly, and very significantly (Wilcoxon p-value <10^-10^) down-regulated. Globally the expression of 27 genes encoding ribosomal proteins as well as 6 genes encoding translation factors and 8 encoding amino-acyl tRNA synthetases decreased (Additional file 3). Eleven translation-related proteins showed significant variations and most of these were decreased (the 3 ribosomal proteins RplJ, RplL and RpsA as well as the transcription factors Frr and Tsf, the amino-acyl tRNA synthetase ArgS, and the protein KsgA also implicated in protein synthesis). In the category "energy metabolism", the expression of genes related to carbon metabolism, more specifically those belonging to “glycolysis” (*enoA*, *ldhX* and *tpiA*), “fermentation” (*ackA1*, *adhE* and *frdC*) and “sugars” (*bglH*, *gntK*, *lacC*, *scrK*, *uxaC*, *uxuB*, *ypcA* and *ypdB*) subcategories, decreased. EnoA, Pyk and YpdD were less abundant too. At the same time, the carbon catabolism repressor, CcpA [29], was down-regulated both at transcript and protein levels. Carbon transport was also affected by isoleucine depletion, since genes linked to “carbohydrate, organic alcohol and acid transport” (*rbsC*, *yngE*, *yngF*, *ypcG*, *ypcH* and *ypdA*) and “PTS transport” (*celB*, *ptcA*, *ptcB*, *ptnAB*, *ptnC*, *ptnD*, *ptsH* and *ptsI*) were under-expressed. Cytoplasmic fractions of the PTS system proteins were detected and the levels of PtnAB and PtsI decreased.

Most of the messengers categorized in “fatty acid metabolism” (*accB*, *accC*, *accD*, *acpA*, *fabF*, *fabG1*, *fabI*, *fabZ1*, *fabZ2*, *fadD*, *lplL*, *plsX* and *thiL*) and “pyrimidine biosynthesis” (*pydB*, *pyrC*, *pyrF*, *pyrG* and *pyrR* encoding the pyrimidine biosynthesis regulator [30]) were significantly under-expressed in response to isoleucine starvation, though these expression decreases were not reflected at the proteome level (Additional files 2 and 3

The decrease of translation, pyrimidine and fatty acid metabolism is consistent with the growth rate reduction observed during isoleucine starvation (see Figure 1B). Similarly the wide down-regulation of carbon transport and metabolism can explain the reduction of glucose consumption rate (see Figure 1B). *L. lactis* ssp. *lactis* adaptation to isoleucine starvation is thus significantly different from *E. coli* response for which over-expression of genes of central metabolism had been observed [12]. The negative control of the catabolism observed in *L. lactis* ssp. *lactis* is likely to adjust metabolic activities to the anabolic demand, thus avoiding energy resource wastage.

#### ii) Specific stress response related to isoleucine starvation

Isoleucine biosynthesis pathway seems to be barely functional in *L. lactis* IL1403. Consistent with this pseudo-auxotrophic phenotype, an extended positive control aiming at supplying cells with isoleucine was observed.

Isoleucine, or BCAA, biosynthesis was above all stimulated by the strong induction of the BCAA biosynthesis pathway (Additional file 3). The expression of the complete *leu-ilv* operon encoding BCAA biosynthesis pathway was strongly increased during isoleucine starvation. With the exception of *ilvA* and *leuA* whose targets were not available on the array, expression ratios in comparison to exponential phase ranged from 1.9 to 5.2 for *ilvB*, *ilvC*, *ilvD*, *ilvN*, *leuB*, *leuC* and *leuD*. The expression of *bcaT*, encoding the amino-transferase catalyzing the last step of BCAA biosynthesis, was also increased significantly. In addition, the level of two proteins from this pathway (IlvD, LeuC) was increased.

CtrA, renamed BcaP, is the main Branched-Chain Amino Acid Permease in *L. lactis* MG1363 (ssp. *cremoris*) [31]. This gene was up-regulated in response to isoleucine starvation (ratio~1.5) but this variation was not statistically significant (FDR~20%). Nevertheless, other amino-acids transporters were up-regulated (*ydgB*, *yjgC*, *yrfD*, *yvdF*) suggesting additional transport possibilities.

**Figure 3:**
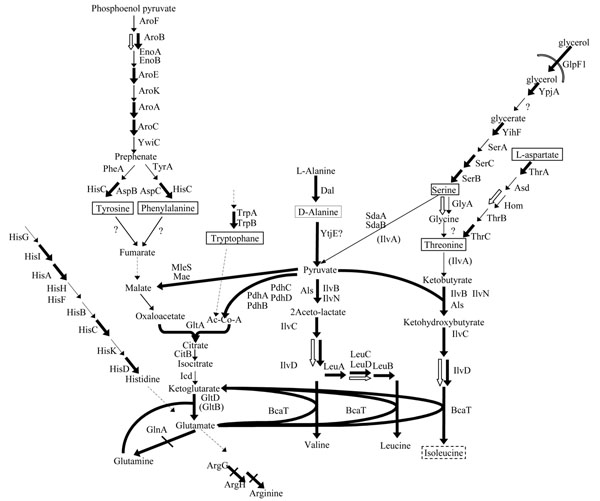
**Coordinated expression of genes and proteins involved in amino acids metabolism during isoleucine starvation in *L. lactis* IL1403.** One arrow represents one metabolic reaction and dashed line arrows correspond to more than one reaction. Protein names, if they are known, are indicated beside the arrows. Brackets stand for genes that are absent from the macro-array. Thick black arrows indicate up-regulation or, if slashed, down-regulation at the transcriptional level. White arrows stand for over-expression of the corresponding protein. Framed amino acids are those that can be synthetised by *L. lactis* IL1403 while the other ones correspond to natural auxotrophies of the strain.

The response of *L. lactis* IL1403 to isoleucine starvation was characterized by a wide and complex metabolic reorganization of the global metabolism of amino acids (Figure 3, Additional file 3). Most of these metabolic adaptations are connected *via* carbon metabolism and converge to the isoleucine biosynthesis pathways. They seem to be dedicated to increase isoleucine production in order to struggle against isoleucine depletion. Firstly, threonine synthesis was favored, probably in order to increase the ketobutyrate pool, which is one of the isoleucine precursors. Direct threonine biosynthesis from aspartate was expected to be enhanced (upregulation of *thrA* and *thrC* and the Hom protein). An alternative pathway of threonine production was also activated from serine/glycine synthesis and catabolism (upregulation of *serB*, *serC*, *yihF* a hypothetical protein sharing protein domain homology with glycerate kinase, *ypjA* an alcohol dehydrogenase, the glycerol uptake facilitator *glpF1* and the GlyA protein). Secondly the synthesis of pyruvate, the other isoleucine precursor, from alanine was also increased (upregulation of *dal* and *ytjE*, respectively encoding a racemase converting D to L alanine and an aminotransferase). Experimental confirmation came from the increase of alanine consumption rate (50 µmol.g^-1^.h^-1^ to 170 µmol.g^-1^.h^-1^ after isoleucine exhaustion) and the detection of traces of pyruvate. Similarly, in *E. coli*, alanine, serine and threonine metabolisms dedicated to isoleucine formation were up-regulated during isoleucine starvation [12]

The gene *gltD*, involved in glutamate biosynthesis from glutamine, which is available in the culture medium, was over-expressed during isoleucine starvation. As a direct consequence, glutamate biosynthesis was enhanced: initially lacking from the culture medium, glutamate was detected after isoleucine starvation and its concentration reached 100 µM at the end of the culture. Since glutamate is also required for BCAA synthesis, this metabolic adaptation can also be considered as a way to increase isoleucine production. Consistent with this metabolic organization favoring glutamate supply, genes *argG*, *argH* and *glnA*, encoding glutamate consuming enzymes for the production of metabolites other than isoleucine, were under-expressed. This response was observed in *L. lactis* ssp. *lactis* but not in *E. coli*. This difference could be related to the lack of glutamate dehydrogenase in LAB and their inability to synthesize glutamate directly from α-ketoglutarate. Other responses, possibly aiming at increasing glutamate supply were observed: the over-expression of genes involved in histidine biosynthetic pathways (*hisA*, *his*C, *hisD* and *hisI*), the over-expression of genes and proteins involved in tyrosine and phenylalanine biosynthesis (*aroA*, *aroB*, *aroC*, *aroD*, *aroE* and AroB protein) and the over-expression of the *gltA* gene encoding citrate synthase and of other genes upstream in carbon metabolism (*mae*, *mleS*, *pdhA*, *pdhB*, *pdhC* and *pdhD*). From a theoretical point of view, a metabolic link could be drawn between these metabolic pathways and glutamate biosynthesis. However, the TCA cycle is considered to be non operative in LAB and histidine biosynthesis to be non-functional in *L. lactis* IL1403. These observed expression changes were probably not able to provide supplementary glutamate and may reflect ancestral regulatory mechanisms of gene expression.

Consistent with this general reorientation of amino acid metabolism, a response at the level of peptides was also observed (Additional file 3). The peptidases encoding genes *htrA*, *pepDA*, *pepO* and *pepXP* were over-expressed. The protein abundance of the peptidase PepO also increased. In addition, genes encoding oligo-peptide transporters (*oppA*, *oppC*, *oppD*, *oppF*, *optA*, *optB*, *optC*, *optD*, *optF* and *optS*) were over-expressed. Lamarque *et al.*[32] did not control the pH of their cultures and failed to detect the *Opp* transcripts in the same strain. This was probably due to acidification since *Opp* is expected to be repressed at low pH [33]. Up-regulation of peptide transport and metabolism in response to isoleucine starvation will favor the assimilation of extra- or intra-cellular peptides and is thus expected to lead to an increase in isoleucine supply.

#### iii) Additional response connected to oxidative stress

The strong decrease of growth and glucose consumption rates when isoleucine was becoming limiting (see Figure 1) demonstrated clearly the high intensity of the stress encountered by starved cells. To avoid the superimposition of natural acidification with isoleucine starvation, pH was maintained at 6.6 (see Methods). Transcriptomic and proteomic analyses did not indicate a general stress response, as was observed when *E. coli* faced isoleucine depletion [12]. Genes belonging to the functional category “adaptation to atypical conditions” (*clpE*, *clpX*, *cpo*, *dinF* and *tpx*) were indeed globally over-expressed (Additional file 1) while the chaperone-encoding genes *dnaK* and *groES* as well as their transcriptional regulator *hrcA*[*34*] were under-expressed. Similarly, at the protein level, the amount of stress-related proteins was increased for ClpE and decreased for GrpE and CspE. In contrast, in *E. coli*, the general stress response was massively up-regulated. This major difference highlights the originality of the *L. lactis* ssp. *lactis* regulation network, which is probably linked to its lack of a stress-induced sigma-like factor.

Isoleucine starvation however provoked the induction of genes and proteins usually related to the oxidative stress response. Genes belonging to the subcategories “thioredoxin, glutaredoxin and glutathione” (*gpo*, *trxB1* and *trxH*) and “detoxification” (*ahpF*) were globally over-expressed (Additional file 3). Correspondingly, the proteins TrxB1 and SodA from these subcategories were increased. Unlike other genes belonging to the category “energy metabolism”, genes involved in “aerobic” subcategory (*cbr*, *yddB*, *ymgK*, *ypaI*, *ypgB*, *yphA*, *yphC*, *ypjA*, *ypjH*, *yrfB*, *yrjC* and *yugC*, but not *noxA* and *noxB*) were also massively over-expressed (Additional file 1) and the protein level of the oxidoreductase YpjH increased strongly. Similarly, the pyruvate dehydrogenase complex expected to be active when oxygen is present in *L. lactis* culture [35] was over-expressed at the transcriptome level (*pdhA*, *pdhB*, *pdhC* and *pdhD*). Finally, “electron transport” subcategory genes (*cydA*, *ndrH*, *ndrI* and *yviC*) were also over-expressed. These oxygen response-like inductions are not related to the presence of oxygen since anaerobic conditions were maintained during the culture (nitrogen atmosphere). Induction of the oxidative stress response has already been observed in *E. coli* at the onset of stationary phase independently of oxygen supply [36]. A link with the reorganization of amino acids metabolism is suggested by the fact that most of these induced genes (*pdhC*, *yddB*, *ymgK*, *ypaI*, *ypgB*, *yphA*, *yphC*, *ypjA*, *ypjF*, *ypjH*, *yrfB*, *yugC*) encode oxidoreductase or dehydrogenase activities involved in amino acid catabolism [37].

### Regulations involved in the isoleucine starvation response

**Figure 4:**
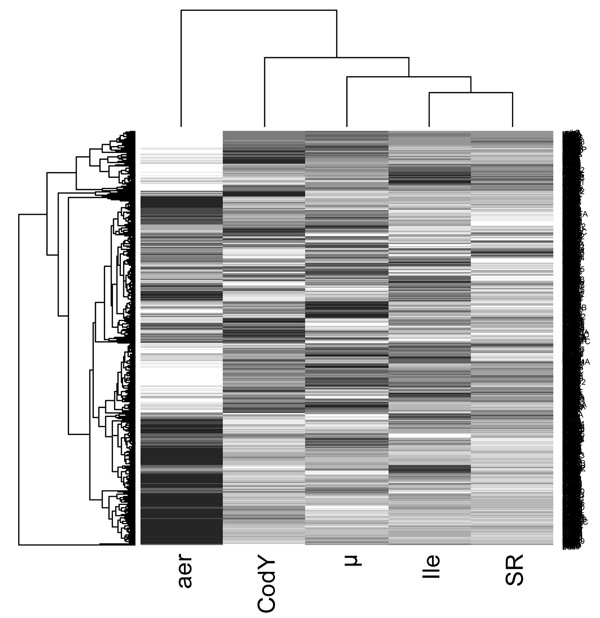
**Double hierarchical clustering to identify gene pattern similarity between various experiments.** Each row correspond to a single gene, the strongest is expression compared to the reference, the darker the color and *vice- versa*. The dendrogramme on top of the figure shows how similar are the compared conditions. Ile = isoleucine starvation response (this study), aer = aeration stimulon [38], µ = growth rate effect [17], SR = stringent response [17].

To further investigate regulations that could occur and coordinate this adaptation process, growth rate and stringent responses [17], CodY regulon [18] and aeration stimulon [38], available in the literature, were compared to our data (additional file 4). It should be noticed that all literature transcriptomic data used in this section were carried out with the same *L. lactis* IL1403 strain except the aeration stimulon performed with the very similar strain CHCC2862 which shares 99.8 % sequence similarity [38].The stringent response and the growth rate related regulation were the major phenomena involved in the isoleucine starvation response with a global overlap of 33 % and 26 % respectively. The overlaps with codY regulon and aeration stimulon were significantly lower with only 7 % and 6 % respectively. Those results were confirmed by the double hierarchical clustering (Figure 4). The comparison of all genes expression values indeed showed a close proximity between stringent and isoleucine starvation responses. The hierarchical distance then increased with growth rate response, CodY regulation, aeration stimulon being the most distant.

Regulations of the three major responses involved in the isoleucine starvation have been elucidated. First, major physiological activities were negatively regulated both by growth rate related and stringent regulations. Under-expression of genes involved in “transcription”, “translation” and “fatty acid metabolism” were, for a majority, related to growth rate regulation, while “pyrimidines” and “carbon metabolism and transport” (“glycolysis”, “fermentation”, “sugars”, “carbohydrate, organic alcohol and acids transport” and “PTS transport” subcategories) were rather linked to the stringent response. Secondly, the response specifically dedicated to cope with isoleucine depletion, including metabolic pathways dedicated to glutamate production, all other amino acid biosynthesis pathways (except for aromatic amino acid) and amino acid and peptide carriers, seemed to be mainly under CodY control. On the other hand, genes involved in aromatic amino acid biosynthesis as well as those encoding peptidases were rather controlled by the stringent mechanism. Lastly, genes involved in the oxygen cross protection and belonging to the subcategories “thioredoxin, glutaredoxin and glutathione”, “detoxification”, “aerobic energy metabolism” and “electron transport pathways” were generally controlled both by stringent response and growth rate related regulators and overlapped only weakly with the oxygen stimulon. Genes classified in “pyruvate dehydrogenase” subcategory were however not regulated by any of these mechanisms. It is known that in *L. lactis* MG1363 these genes are under CcpA control [29]. The potential involvement of CcpA control in the isoleucine starvation would agree with results obtained for *B. subtilis*[10].

## Conclusions

A sound description of the response to isoleucine starvation was obtained through a fermentation study coupled with proteomic and transcriptomic analyses in the bacterium model *L. lactis* IL1403. It has extended the physiological understanding of the metabolism of this bacterium. A regulatory network more complex than previously thought, linking nitrogen and carbon metabolism and involving various regulations, was revealed. The response was found to occur gradually with the involvement of more and more genes and proteins during the dynamic. The isoleucine starvation response could be divided into three main mechanisms. Firstly, a reduction of major physiological activities, presumably regulated both by growth rate and stringent response associated regulators, was observed. Secondly, a response specifically dedicated to cope with the imposed starvation was defined. This process seemed to be under stringent and CodY control. Lastly, a response related to oxidative stress controlled by the growth rate related regulator and stringent response was described. Finally, growth rate related and stringent responses were, by far, the global regulatory mechanisms mostly involved in isoleucine starvation response. The implementation of such an integrated and comparative approach to other regulations and environmental conditions, will allow the whole regulatory network of *L. lactis* to be deciphered.

## Competing interests

The authors declare that they have no competing interests.

## Supplementary Material

Additional file 1 Table S1 Click here for file

Additional file 2 Table S2 Click here for file

Additional file 3 Table S3. Transcriptomic and proteomic responses of *L. lactis* IL1403 to isoleucine starvation. Click here for file

Additional file 4 Table S4 Click here for file
