# Supplementary material for: Investigation of the adaptation of Lactococcus lactis to isoleucine starvation integrating dynamic transcriptome and proteome information
Source: Microb Cell Fact. 2011 Aug 30;10(Suppl 1):S18. doi: 10.1186/1475-2859-10-S1-S18 (PMC3236307; doi:10.1186/1475-2859-10-S1-S18)
Supplement: Additional file 3 — Table S3. Transcriptomic and proteomic responses of L. lactis IL1403 to isoleucine starvation. [file 1475-2859-10-S1-S18-S3.doc]

**Additional file 1: Table S3.** Transcriptomic and proteomic responses of *L. lactis* IL1403 to isoleucine starvation (three independent repetitions)

List of transcripts and proteins commented in the main text and significantly up or down regulated 20 min, 1.34 h and 3 h after isoleucine exhaustion compared to the exponential phase and classified as defined by Bolotin . Ratio values are indicated as exponent and data are ranged by decreasing ratios. Signs + or – followed by a number in brackets represent respectively significant up or down-regulation and the associated Wilcoxon p-value of the functional (sub) category according to Wilcoxon test.

|  | TRANSCRIPTOME | | | PROTEOME | | |
| --- | --- | --- | --- | --- | --- | --- |
| FUNCTIONNAL CATEGORIES or sub-categories | 20 min | 1.34 h | 3.00 h | 20 min | 1.34 h | 3.00 h |
| **AMINO ACID BIOSYNTHESIS** | **+ (1.2x10^-4^)** | **+ (3.3x10^-5^)** | **+ (2.1x10^-4^)** |  |  |  |
| Aromatic amino acid family | **+ (2.8x10^-2^)**  aroC^1.88^, aroA^1.57^, aroE^1.49*^, aroH^0.7^ | **+ (1.9x10^-2^)**  aroC^1.7^, aroA^1.39^, aroD^1.37^, aroH^0.43^ | **+ (7.8x10^-3^)**  trpA^1.77^, trpB^1.76^, aroC^1.62^, aroA^1.38^, aroB^1.37^, aroD^1.26^, aroH^0.38^ | AroB^1.7^ | AroB^2.81^ |  |
| Aspartate family | **+ (1.7x10^-2^)**  dapA^1.51^ | **+ (1.8x10^-2^)**  thrA^2.08^, dapA^1.7^ | **+ (2.9x10^-2^)**  thrA^2.08^, dapA^1.85^, thrC^1.29^ | Hom^1.4^ | LysA^2.07^ | Hom^1.98^ |
| Branched chain family | **+ (9.7x10^-5^)**  leuD^6.8^, leuC^6.35^, ilvD^4.71^, ilvB^3.57^, leuB^3.24^, ilvN^3.23^, ilvC^2.2^ | **+ (3.0x10^-5^)**  leuC^4.95^, leuD^4.93^, ilvD^3.85^, leuB^2.58^, ilvN^2.5^, ilvB^2.28^ | **+ (5.4x10^-4^)**  leuD^3.8^, leuC^3.74^, ilvD^2.96^, leuB^2.04^ | IlvD^2.56^, LeuC^2.5^ |  | IlvD^4.74^ |
| Glutamate family | gltD^1.77*^, gltA^1.35^, glnA^0.5^ | argG^0.74^, argH^0.6^, glnA^0.28^ | argG^0.76^, argH^0.55^, glnA^0.29^ |  |  |  |
| Histidine family | **+ (4.2x10^-2^)**  hisI^1.54^, hisA^1.5^, hisC^1.31^ | **+ (7.2x10^-3^)**  hisI^1.59^, hisC^1.5^, hisA^1.44^, hisD^1.4^ | **+ (3.5x10^-2^)**  hisC^1.64^, hisD^1.56^, hisA^1.52^, hisI^1.48^ |  |  |  |
| Serine family | serB^1.9^, serC^1.63^ | serB^1.71^, serC^1.47^ | serB^1.73^, serC^1.43^ |  | GlyA^2.59^ | GlyA^2.1^ |
| **BIOSYNTHESIS OF COFACTORS** |  |  |  |  |  |  |
| Thioredoxin, glutaredoxin and glutathione | **+ (1.0x10^-2^)**  trxB1^1.84^ | **+ (2.9x10^-3^)**  trxB1^2.26^, trxH^1.58^ | **+ (1.9x10^-3^)**  trxB1^2.6^, trxH^1.65^, gpo^1.64^ |  | TrxB1^2.02^ | TrxB1^2.44^ |
| **CELLULAR PROCESS** |  |  |  |  |  |  |
| Cell division | rodA^0.57^ | gidB^0.68^, rodA^0.48^ | gidB^0.69^, rodA^0.48^ |  |  |  |
|  |  |  |  |  |  |  |
| Chaperones | **- (4.0x10^-2^)**  groES^0.64^, dnaK^0.48^ | **- (2.0x10^-2^)**  groES^0.69^, dnaK^0.47^ | dnaK^0.53^ |  |  |  |
| Detoxification | **+ (1.9x10^-2^)**  ahpF^1.78^ | **+ (1.9x10^-2^)**  ahpF^2^ | **+ (2.9x10^-2^)**  ahpF^2.23^ |  |  | SodA^1.79^ |
| **ENERGY METABOLISM** | **- (1.5x10^-2^)** |  |  |  |  |  |
| Aerobic | ypjH^2.93^, yphA^2.39^, yphC^2.19^, yugC^1.87^, ymgK^1.68^, yrfB^1.54^, ypaI^1.34^, ypjA^1.33^, noxB^0.49^ | **+ (2.2x10^-3^)**  ypjH^2.74^, yphA^2.44^, yphC^1.96^, yrfB^1.85^, ymgK^1.81^, yugC^1.74^, ypgB^1.48^, ypaI^1.44^, yddB^1.43^, ypjA^1.23^, noxC^1.22^, ycdG^0.69^, noxA^0.52^, noxB^0.41^ | **+ (5.6x10^-3^)**  yphA^2.43^, ypjH^2.37^, yphC^2.32^, yrfB^2.15^, yugC^1.88^, ybgA^1.84^, ymgK^1.75^, cbr^1.54^, ypaI^1.46^, yrjC^1.39^, ypjA^1.31^, ycdG^0.66^, noxA^0.55^, noxB^0.39^ | YpjH^8.81^ | YpjH^15.55^ | YpjH^17.81^ |
| Electron transport | **+ (5.4x10^-3^)**  ndrH^2.35^, ndrI^2^, yviC^1.38^ | **+ (3.7x10^-2^)**  ndrH^2.65^, ndrI^2.65^, fer^0.76^ | **+ (1.9x10^-2^)**  ndrH^2.66^, ndrI^2.44^, yviC^1.49^, cydA^1.32^, yfjE^0.73^, fer^0.72^ |  |  |  |
| Fermentation | **- (6.0x10^-3^)**  aldB^3.76^, ackA1^0.74^, frdC^0.68^, adhE^0.15^ | **- (3.5x10^-2^)**  aldB^3.03^, mleS^1.39^, ackA1^0.67^, frdC^0.59^, adhE^0.14^ | aldB^2.78^, mleS^1.57^, mae^1.19^, ackA1^0.69^, frdC^0.62^, adhE^0.15^ |  |  |  |
| Glycolysis | ldhX^0.59^, enoA^0.56^ | **- (1.6x10^-2^)**  ldhX^0.58^, enoA^0.5^ | **- (2.7x10^-2^)**  tpiA^0.76^, ldhX^0.55^, enoA^0.49^ |  | Pyk^0.86^, EnoA^0.74^ | EnoA^0.66^ |
| Pyruvate dehydrogenase | **+ (8.2x10^-3^)**  pdhD^1.74^ | **+ (1.6x10^-2^)**  pdhD^1.66^, pdhC^1.58^ | **+ (1.8x10^-2^)**  pdhD^1.67^, pdhA^1.48^, pdhC^1.48^, pdhB^1.28^ |  |  |  |
| Sugars | **- (4.8x10^-3^)**  uxuB^0.7^, uxaC^0.49^, bglH^0.46^, scrK^0.38^, ypdB^0.35^, ypcA^0.24^ | **- (3.2x10^-2^)**  lacC^0.73^, bglH^0.48^, scrK^0.39^, ypdB^0.36^, ypcA^0.27^ | **- (4.7x10^-2^)**  xylX^2.01^, galK^1.69^, galM^1.28^, gntK^0.73^, pmi^0.69^, bglH^0.62^, scrK^0.44^, ypdB^0.34^, ypcA^0.21^ |  | YpdD^0.73^ |  |
| TCA cycle | citC^0.81^ |  | citC^0.78^ |  |  |  |
| **FATTY ACID AND PHOSPHOLIPID METABOLISM** | **- (4.6x10^-6^)**  fbaI^0.76^, plsX^0.67^, accC^0.55^, accD^0.55^, fabF^0.52^, fabG1^0.46^, accB^0.43^, fadD^0.26^ | **- (3.0x10^-6^)**  plsX^0.79^, lplL^0.63^, fabZ1^0.49^, accD^0.4^, acpA^0.4^, fadD^0.4^, accC^0.38^, fabF^0.36^, fabZ2^0.33^, accB^0.31^, fabG1^0.29^ | **- (1.6x10^-6^)**  acpD^1.43^, fabI^0.78^, plsX^0.76^, lplL^0.67^, thiL^0.62^, fabZ1^0.53^, fadD^0.41^, acpA^0.38^, fabF^0.38^, accC^0.37^, accD^0.37^, fabZ2^0.32^, accB^0.31^, fabG1^0.27^ |  | AccC^1.54^, FabF^1.35^ | ThiL^2.99^, YdiD^2.26^ |
|  |  |  |  |  |  |  |
| **PURINES, PYRIMIDINES, NUCLEOSIDES AND NUCLEOTIDES** |  |  | **- (4.6x10^-2^)** |  |  |  |
| Purine ribonucleotide biosynthesis | guaB^1.87^ | guaB^2.15^, purM^1.1^, purN^1.1^, guaC^0.45^ | guaB^2.42^, guaA^1.4^, guaC^0.5^ | PurM^21.38^ , PurL^11.71^, PurH^6.05^, PurB^1.39^, | PurM^27.98^, Pur^9.93^, PurH^5.67^, PurB^1.51^ | PurM^27.01^, PurL^9.85^, PurH^5.32^ |
| Pyrimidine ribonucleotide biosynthesis |  | **- (3.0x10^-2^)**  pyrG^0.54^ | **- (1.7x10^-2^)**  thyA^1.37^, pydB^0.68^, pyrC^0.66^, pyrG^0.56^, pyrF^0.42^ | PyrB^2.54^, ThyA^2.47^, PyrC^1.86^ | ThyA^3^, PyrB^2.63^, PyrC^1.82^ | ThyA^2.5^, PyrB^2.23^, PyrC^1.71^ |
| **TRANSCRIPTION** |  |  |  |  |  |  |
| Degradation of RNA | vacB1^1.93^ | vacB1^1.68^ | vacB1^1.81^, vacB2^1.6^ |  |  |  |
| RNA synthesis, modification and DNA transcription | rpoD^1.72^, rpoB^0.54^ | rpoD^1.7^, yfjD^1.61^, rsuA^0.8^, RpoA^0.75^, rpoB^0.47^ | rpoD^1.81^, yfjD^1.77^, nusG^1.64^, rsuA^0.73^, rpoA^0.71^, rpoC^0.6^, rpoB^0.44^ |  |  |  |
| RNA processing |  | rimM^0.78^ | rheB^01.28^ |  |  |  |
| **TRANSLATION** | **- (6.3x10^-13^)** | **- (5.5x10^-15^)** | **- (5.3x10^-16^)** |  |  |  |
| Amino acyl tRNA synthetases | **- (1.7x10^-3^)**  ileS^2.06^, proS^1.2^, glyS^0.75^, argS^0.65^, pheS^0.55^, thrS^0.48^ | **- (5.5x10^-4^)**  ileS^1.9^, argS^0.76^, alaS^0.74^, valS^0.68^, glyS^0.63^, pheS^0.51^, thrS^0.5^ | **- (3.0x10^-4^)**  ileS^1.94^, alaS^0.8^, argS^0.79^, lysS^0.76^, glyS^0.71^, pheS^0.52^, thrS^0.49^, tyrS^0.45^ | GltX^2.32^, ArgS^0.84^ | TyrS^1.29^, ArgS^0.87^ | TyrS^1.27^ |
| Degradation of proteins, peptides and glycopeptides | pepXP^1.93^, pepO^1.56^, htrA^1.43^ | pepXP^1.86^, pepDA^1.47^, htrA^1.41^, yueF^0.63^ | pepXP^2.09^, pepDA^1.52^, pepO^1.49^, htrA^1.35^, yueF^0.59^ |  | PepO^1.58^ | PepO^1.78^ |
| Protein modification | pmsR^1.89^, ytaD^1.4^ | ytaD^1.66^, pmsR^1.54^ | pmsR^1.84^, ytaD^1.82^ |  |  |  |
| Ribosomal proteins: synthesis and modification | **- (1.2.x10^-11^)**  rpmGA^1.45^, rpsP^1.39^, gatB^1.34^, rplQ^0.74^, rpsK^0.74^, rpsG^0.63^, rpsS^0.61^, rpsU^0.61^, rplN^0.6^, rpsJ^0.6^, rpmB^0.59^, rpsQ^0.59^, rpsI^0.58^, rpsC^0.55^, rpsL^0.55^, rpmA^0.54^, rpmI^0.52^ | **- (1.3.x10^-11^)**  prmA^0.76^, rplQ^0.7^, rpsK^0.7^, rpsG^0.67^, rpsC^0.62^, rpsB^0.6^, rplS^0.57^, yhdC^0.56^, rpsI^0.55^, rpsJ^0.55^, rplN^0.54^, rpmB^0.54^, rpsQ^0.54^, rplB^0.53^, rpsS^0.53^, rpsF^0.51^, rpsU^0.5^, rplM^0.46^, rpsL^0.46^, rpmI^0.39^ | **- (1.8.x10^-13^)**  trmU^1.58^, yhjG^1.53^, fmt^1.29^, rplQ^0.83^, prmA^0.81^, rplO^0.71^, rpsK^0.69^, rpsG^0.66^, rpsB^0.63^, yhdC^0.62^, rpsR^0.6^, rplS^0.59^, rpsQ^0.57^, rplB^0.56^, rpsC^0.56^, rpsJ^0.56^, rpsI^0.55^, rpsS^0.55^, rplN^0.52^, rpsU^0.52^, rplU^0.51^, rpmB^0.5^, rpsF^0.49^, rpsL^0.47^, rplT^0.46^, rplM^0.44^, rplV^0.43^, rpmI0^.4^, rpsH^0.39^ | RplJ^0.81^, RpsA^0.75^, KsgA^0.5^ | RplJ^0.76^, RpsA^0.71^ | RpmC^1.29^, RpsA^0.66^,  RplL^0.55^ |
| Translation factors | **- (3.0x10^-3^)**  efp^0.75^, infA^0.67^, tsf^0.66^, fusA^0.65^, infC^0.59^ | **- (8.1x10^-4^)**  infA^0.65^, fusA^0.64^, tsf^0.63^, infC^0.52^ | **- (6.9x10^-4^)**  infB^0.78^, fusA^0.65^, infA^0.6^, tsf^0.59^, infC^0.5^ | Tsf^0.83^, Frr^0.76^ |  | Tsf^0.76^ |
| **TRANSPORT AND BINDING PROTEINS** |  | **- (3.3x10^-2^)** | **- (1.9x10^-2^)** |  |  |  |
| Amino acids, peptides and amines | **+ (3.0x10^-3^)**  optS^3.27^, busAA^3.24^, busAB^2.99^, oppA^2.44^, optC^2.21^, oppD^2.11^, optD^2.11^, yjgC^2.1^, optB^1.83^, optA^1.82^, oppF^1.73^, ydgB^1.42^, yfcG^1.29^, glnP^0.76^, ylcA^0.62^, lysP^0.47^ | **+ (2.9x10^-2^)**  busAA^4.84^, busAB^5.2^, optS^2.48^, oppA^2.46^, oppD^2.42^, yjgC^2.25^, optD^2.19^, optC^2.13^, oppC^2.07^, yvdF^2.05^, optF^1.99^, oppF^1.92^, optB^1.74^, optA^1.72^, yfcG^1.45^, ydgB^1.4^, glnP^0.66^, ylcA^0.58^, lysP^0.49^ | busAB^5.42^, busAA^5.1^, yjgC^2.88^, oppD^2.42^, yvdF^2.29^, oppA^2.26^, oppC^2.16^, optC^2.1^, optD^2.03^, optS^1.91^, oppF^1.75^, optB^1.71^, optA^1.64^, ydgB^1.44^, yrfD^1.44^, yfcG^1.36^, choS^1.22^, arcD1^1.14^, yshA^0.77^, glnP^0.64^, lysP^0.61^, ylcA^0.56^ |  |  |  |
|  |  |  |  |  |  |  |
| Carbohydrates, organic alcohols and acids | **- (2.4x10^-4^)**  glpF1^1.26^, ypdA^0.41^, yphC^0.35^, ypcG^0.26^ | **- (2.6x10^-4^)**  ypbD^1.49^, yqgE^1.27^, yngF^0.63^, ypcH^0.35^, ypdA^0.34^, ypcG^0.27^ | **- (2.5x10^-3^)**  ypbD^1.54^, yqgE^1.41^, glpF1^1.3^, rbsC^0.75^, yngE^0.61^, yngF^0.55^, ypcH^0.34^, ypdA^0.33^, ypcG^0.23^ |  |  |  |
| PTS system | **- (2.4x10^-5^)**  ptsI^0.73^, ptsH^0.57^, ptnD^0.43^, ptcA^0.42^, ptnAB^0.39^, ptnC^0.34^ | **- (4.9x10^-4^)**  ptsI^0.82^, celB^0.6^, ptsH^0.59^, ptcA^0.4^, ptnD^0.36^, ptnAB^0.32^, ptnC^0.28^ | **- (5.0x10^-5^)**  ptsI^0.79^, ptsH^0.58^, ptcB^0.48^, ptnD^0.44^, ptcA^0.41^, ptnC^0.28^, ptnAB^0.27^ | PtnAB^0.65^ |  | PtsI^0.69^ |
| **OTHER CATEGORIES** | **- (6.2x10^-6^)** |  |  |  |  |  |
| Adaptations and atypical conditions | cpo^1.35^, hrcA^0.62^ | **+ (4.8x10^-2^)**  clpE^1.58^, cpo^1.47^, tpx^1.39^, clpX^1.18^, hrcA^0.6^ | clpE^1.67^, tpx^1.48^, cpo^1.42^, dinF^1.4^, clpX^1.3^, hrcA^0.67^ | ClpE^3.13^, CspE^0.59^, GrpE^0.5^ | GrpE^0.67^ | GrpE^0.49^ |

* genes differentially expressed but with FDR hardly higher than the significativity threshold.
