# Supplementary material for: Investigation of the adaptation of Lactococcus lactis to isoleucine starvation integrating dynamic transcriptome and proteome information
Source: Microb Cell Fact. 2011 Aug 30;10(Suppl 1):S18. doi: 10.1186/1475-2859-10-S1-S18 (PMC3236307; doi:10.1186/1475-2859-10-S1-S18)
Supplement: Additional file 4 — Table S4 [file 1475-2859-10-S1-S18-S4.doc]

**Table S4:** Comparison of *L. lactis* IL1403 response to isoleucine starvation with other responses available in the literature.

Signs + or - represents respectively genes up- or down- regulated. Numbers in the column entitled Ile represent genes involved in each (sub) category during isoleucine starvation response. Other columns give the number of genes that are common between isoleucine starvation response and the given response: growth rate decrease (µ), stringent response induction (SR), *codY* (CodY), *ccpA* or *glnR* (GlnR) deletion and aeration stress (O_2_).

| FUNCTIONAL CATEGORIES  or sub-categories | + | | | | | - | | | | |
| --- | --- | --- | --- | --- | --- | --- | --- | --- | --- | --- |
|  | Ile | µ | SR | CodY | O_2_ | Ile | µ | SR | CodY | O_2_ |
| AMINO ACID BIOSYNTHESIS | 25 | 2 | 8 | 17 | 0 | 4 | 2 | 1 | 0 | 0 |
| -- Aromatic amino acid family | 7 | 0 | 3 | 0 | 0 | 1 | 1 | 0 | 0 | 0 |
| -- Aspartate family | 3 | 0 | 1 | 2 | 0 | 0 | 0 | 0 | 0 | 0 |
| -- Branched chain family | 7 | 0 | 3 | 7 | 0 | 0 | 0 | 0 | 0 | 0 |
| -- Glutamate family | 2 | 0 | 0 | 2 | 0 | 3 | 1 | 1 | 0 | 0 |
| -- Histidine family | 4 | 2 | 1 | 4 | 0 | 0 | 0 | 0 | 0 | 0 |
| -- Serine family | 2 | 0 | 0 | 2 | 0 | 0 | 0 | 0 | 0 | 0 |
| BIOSYNTHESIS OF COFACTORS, PROSTHETIC GROUPS, AND CARRIERS | 6 | 1 | 3 | 0 | 1 | 11 | 0 | 2 | 0 | 0 |
| -- Folic acid | 1 | 0 | 1 | 0 | 0 | 2 | 0 | 0 | 0 | 0 |
| -- Heme and porphyrin | 0 | 0 | 0 | 0 | 0 | 0 | 0 | 0 | 0 | 0 |
| -- Menaquinone and ubiquinone | 0 | 0 | 0 | 0 | 0 | 5 | 0 | 1 | 0 | 0 |
| -- Pantothenate | 1 | 0 | 0 | 0 | 0 | 2 | 0 | 0 | 0 | 0 |
| -- Riboflavin and cobalamin | 1 | 1 | 0 | 0 | 0 | 1 | 0 | 0 | 0 | 0 |
| -- Thioredoxin, glutaredoxin and glutathione | 3 | 0 | 2 | 0 | 1 | 0 | 0 | 0 | 0 | 0 |
| -- Thiamin | 0 | 0 | 0 | 0 | 0 | 1 | 0 | 1 | 0 | 0 |
| -- Pyridine nucleotides | 0 | 0 | 0 | 0 | 0 | 0 | 0 | 0 | 0 | 0 |
| CELL ENVELOPE | 12 | 3 | 4 | 0 | 2 | 10 | 4 | 2 | 0 | 1 |
| -- Membranes, lipoproteins and porins | 1 | 0 | 0 | 0 | 0 | 4 | 1 | 1 | 0 | 1 |
| -- Murein sacculus and peptidoglycan | 6 | 0 | 2 | 0 | 0 | 2 | 0 | 1 | 0 | 0 |
| -- Surface polysaccharides and antigens | 5 | 3 | 2 | 0 | 2 | 4 | 3 | 0 | 0 | 0 |
| CELLULAR PROCESSES | 4 | 1 | 1 | 1 | 1 | 3 | 1 | 1 | 0 | 0 |
| -- Cell division | 0 | 0 | 0 | 0 | 0 | 1 | 0 | 0 | 0 | 0 |
| -- Cell killing | 0 | 0 | 0 | 0 | 0 | 0 | 0 | 0 | 0 | 0 |
| -- Chaperones | 0 | 0 | 0 | 0 | 0 | 2 | 1 | 1 | 0 | 0 |
| -- Detoxification | 1 | 1 | 1 | 1 | 1 | 0 | 0 | 0 | 0 | 0 |
| -- Protein and peptide secretion | 1 | 0 | 0 | 0 | 0 | 0 | 0 | 0 | 0 | 0 |
| -- Transformation | 2 | 0 | 0 | 0 | 0 | 0 | 0 | 0 | 0 | 0 |
| CENTRAL INTERMEDIARY METABOLISM | 4 | 0 | 2 | 2 | 2 | 4 | 0 | 3 | 0 | 0 |
| -- General | 0 | 0 | 0 | 0 | 0 | 0 | 0 | 0 | 0 | 0 |
| FUNCTIONAL CATEGORIES  or sub-categories | + | | | | | - | | | | |
|  | Ile | µ | SR | CodY | O_2_ | Ile | µ | SR | CodY | O_2_ |
| -- Amino sugars | 2 | 0 | 0 | 0 | 0 | 2 | 0 | 1 | 0 | 0 |
| -- Degradation of polysaccharides | 2 | 0 | 1 | 1 | 1 | 2 | 0 | 2 | 0 | 0 |
| -- Phosphorus compounds | 0 | 0 | 0 | 0 | 0 | 0 | 0 | 0 | 0 | 0 |
| -- Polyamine biosynthesis | 0 | 0 | 0 | 0 | 0 | 0 | 0 | 0 | 0 | 0 |
| -- Other | 0 | 0 | 1 | 1 | 1 | 0 | 0 | 0 | 0 | 0 |
| ENERGY METABOLISM | 35 | 10 | 16 | 4 | 6 | 25 | 6 | 9 | 0 | 1 |
| -- Aerobic | 12 | 5 | 6 | 1 | 1 | 3 | 2 | 1 | 0 | 0 |
| -- Amino acids and amines | 6 | 2 | 3 | 1 | 0 | 1 | 0 | 0 | 0 | 1 |
| -- Anaerobic | 0 | 0 | 0 | 0 | 0 | 0 | 0 | 0 | 0 | 0 |
| -- ATP-PMF conversion | 0 | 0 | 0 | 0 | 0 | 0 | 0 | 0 | 0 | 0 |
| -- Electron transport | 4 | 1 | 2 | 0 | 1 | 2 | 0 | 0 | 0 | 0 |
| -- Entner-Doudoroff | 0 | 0 | 0 | 0 | 0 | 0 | 0 | 0 | 0 | 0 |
| -- Fermentation | 3 | 0 | 1 | 2 | 0 | 3 | 1 | 1 | 0 | 0 |
| -- Gluconeogenesis | 0 | 0 | 0 | 0 | 0 | 1 | 0 | 0 | 0 | 0 |
| -- Glycolysis | 0 | 0 | 0 | 0 | 0 | 3 | 2 | 1 | 0 | 0 |
| --Pentose phosphate pathway | 3 | 1 | 2 | 0 | 1 | 2 | 2 | 0 | 0 | 0 |
| -- Pyruvate dehydrogenase | 4 | 0 | 1 | 0 | 3 | 0 | 0 | 0 | 0 | 0 |
| -- Sugars | 3 | 1 | 1 | 0 | 0 | 9 | 0 | 6 | 0 | 0 |
| -- TCA cycle | 0 | 0 | 0 | 0 | 0 | 1 | 1 | 0 | 0 | 0 |
| FATTY ACID AND PHOSPHOLIPID METABOLISM | 1 | 0 | 0 | 0 | 0 | 13 | 9 | 2 | 0 | 0 |
| PURINES, PYRIMIDINES, NUCLEOSIDES AND NUCLEOTIDES | 13 | 3 | 4 | 0 | 2 | 10 | 3 | 6 | 0 | 0 |
| -- 2'-deoxyribonucleotide metabolism | 2 | 0 | 2 | 0 | 0 | 0 | 0 | 0 | 0 | 0 |
| -- Nucleotide and nucleoside interconversion | 2 | 0 | 0 | 0 | 1 | 1 | 0 | 0 | 0 | 0 |
| -- Purine ribonucleotide biosynthesis | 4 | 2 | 2 | 0 | 0 | 2 | 0 | 2 | 0 | 0 |
| -- Pyrimidine ribonucleotide biosynthesis | 1 | 0 | 0 | 0 | 0 | 3 | 1 | 3 | 0 | 0 |
| -- Salvage of nucleosides and nucleotides | 4 | 1 | 0 | 0 | 1 | 3 | 1 | 1 | 0 | 0 |
| -- Sugar-nucleotide biosynthesis and interconversions | 0 | 0 | 0 | 0 | 0 | 1 | 1 | 0 | 0 | 0 |
| REGULATORY FUNCTIONS | 27 | 2 | 8 | 2 | 1 | 14 | 4 | 3 | 0 | 0 |
| -- General | 18 | 1 | 5 | 2 | 1 | 6 | 2 | 3 | 0 | 0 |
| -- Two-component systems | 1 | 0 | 0 | 0 | 0 | 2 | 0 | 0 | 0 | 0 |
| -- Lac-Ifamily regulators | 1 | 0 | 1 | 0 | 0 | 1 | 0 | 0 | 0 | 0 |
| -- LysR-family regulators | 2 | 0 | 0 | 0 | 0 | 0 | 0 | 0 | 0 | 0 |
| -- AraC-family regulators | 1 | 0 | 1 | 0 | 0 | 0 | 0 | 0 | 0 | 0 |
| -- GntR-family regulators | 0 | 0 | 0 | 0 | 0 | 1 | 0 | 0 | 0 | 0 |
| -- DeoR-family regulators | 0 | 0 | 0 | 0 | 0 | 1 | 0 | 0 | 0 | 0 |
| -- MarR-family regulators | 3 | 1 | 1 | 0 | 0 | 3 | 2 | 0 | 0 | 0 |
| FUNCTIONAL CATEGORIES  or sub-categories | + | | | | | - | | | | |
|  | Ile | µ | SR | CodY | O_2_ | Ile | µ | SR | CodY | O_2_ |
| -- BglG-family regulators | 0 | 0 | 0 | 0 | 0 | 0 | 0 | 0 | 0 | 0 |
| -- GTP-binding proteins | 1 | 0 | 0 | 0 | 0 | 0 | 0 | 0 | 0 | 0 |
| REPLICATION | 9 | 1 | 2 | 0 | 0 | 2 | 0 | 1 | 0 | 0 |
| -- Degradation of DNA | 2 | 0 | 0 | 0 | 0 | 1 | 0 | 0 | 0 | 0 |
| -- DNA replication, restriction, modification, recombination and repair | 7 | 1 | 2 | 0 | 0 | 1 | 0 | 1 | 0 | 0 |
| TRANSCRIPTION | 6 | 0 | 0 | 0 | 0 | 5 | 3 | 0 | 0 | 0 |
| -- Degradation of RNA | 2 | 0 | 0 | 0 | 0 | 0 | 0 | 0 | 0 | 0 |
| -- RNA synthesis, modification and DNA transcription | 3 | 0 | 0 | 0 | 0 | 4 | 3 | 0 | 0 | 0 |
| -- RNA processing | 1 | 0 | 0 | 0 | 0 | 1 | 0 | 0 | 0 | 0 |
| TRANSLATION | 14 | 3 | 5 | 0 | 1 | 43 | 33 | 11 | 0 | 0 |
| -- Amino acyl tRNA synthetases | 2 | 0 | 1 | 0 | 0 | 8 | 4 | 2 | 0 | 0 |
| -- Degradation of proteins, peptides and glycopeptides | 4 | 1 | 2 | 0 | 0 | 1 | 0 | 1 | 0 | 0 |
| -- Protein modification | 2 | 1 | 1 | 0 | 0 | 0 | 0 | 0 | 0 | 0 |
| -- Ribosomal proteins: synthesis and modification | 6 | 1 | 1 | 0 | 1 | 27 | 26 | 7 | 0 | 0 |
| -- Translation factors | 0 | 0 | 0 | 0 | 0 | 7 | 3 | 1 | 0 | 0 |
| TRANSPORT AND BINDING PROTEINS | 58 | 10 | 3 | 12 | 4 | 32 | 6 | 17 | 1 | 0 |
| -- General | 19 | 0 | 8 | 2 | 1 | 5 | 0 | 1 | 0 | 0 |
| -- Amino acids, peptides and amines | 18 | 4 | 11 | 10 | 2 | 5 | 1 | 3 | 1 | 0 |
| -- Anions | 0 | 0 | 0 | 0 | 0 | 1 | 1 | 0 | 0 | 0 |
| -- Carbohydrates, organic alcohols and acids | 3 | 1 | 2 | 0 | 0 | 6 | 0 | 3 | 0 | 0 |
| -- Cations | 8 | 3 | 4 | 0 | 1 | 5 | 1 | 2 | 0 | 0 |
| -- Nucleosides, purines and pyrimidines | 2 | 0 | 1 | 0 | 0 | 0 | 0 | 0 | 0 | 0 |
| -- PTS system | 0 | 0 | 0 | 0 | 0 | 8 | 3 | 6 | 0 | 0 |
| -- Multidrug resistance | 8 | 2 | 3 | 0 | 0 | 2 | 0 | 2 | 0 | 0 |
| OTHER CATEGORIES | 39 | 18 | 8 | 1 | 2 | 7 | 0 | 1 | 0 | 0 |
| -- Adaptations and atypical conditions | 5 | 1 | 3 | 0 | 2 | 1 | 0 | 1 | 0 | 0 |
| -- Drug and analog sensitivity | 2 | 0 | 0 | 1 | 0 | 0 | 0 | 0 | 0 | 0 |
| -- Phage related functions and prophages | 27 | 12 | 4 | 0 | 0 | 6 | 0 | 0 | 0 | 0 |
| -- Transposon related functions | 4 | 4 | 1 | 0 | 0 | 0 | 0 | 0 | 0 | 0 |
| -- Other | 1 | 1 | 0 | 0 | 0 | 0 | 0 | 0 | 0 | 0 |
| UNKNOWN | 152 | 27 | 56 | 4 | 12 | 79 | 25 | 16 | 1 | 2 |
| **Total (in number of genes)** | **405** | **81** | **146** | **43** | **34** | **262** | **96** | **75** | **2** | **4** |
| **Global overlapping**  **(in percentage of Ile regulon)** | **61** | **12** | **22** | **6** | **5** | **39** | **14** | **11** | **<1** | **1** |
